# Proximal and Long-Term Participants’ Perspectives on Approach Bias Modification for Smoking Cessation: Qualitative Study

**DOI:** 10.2196/93238

**Published:** 2026-06-22

**Authors:** Alla Machulska, Johanna Bück, Esra Sünkel, Marie Neubert, Tim Klucken

**Affiliations:** 1 Clinical Psychology and Psychotherapy Department of Psychology University of Siegen Siegen, North Rhine-Westphalia Germany

**Keywords:** approach bias modification, cognitive bias modification, long-term outcomes, mobile app, qualitative analysis, smoking cessation, subjective experiences, virtual reality

## Abstract

**Background:**

Approach bias modification (ApBM) aims to target maladaptive approach tendencies toward substance-related cues and has increasingly been examined as an adjunctive intervention for substance use disorders, including nicotine use. However, although participants’ subjective experiences of ApBM are likely to influence both its effectiveness and successful implementation, they have rarely been systematically investigated.

**Objective:**

This study aimed to explore both proximate and long-term subjective training experiences reported by participants from 2 previously conducted randomized controlled trials of ApBM for smoking cessation. Training experiences were explored across 2 delivery modalities (virtual reality and smartphone app) and compared with those reported in matched sham training conditions.

**Methods:**

Participants were invited to provide open-ended feedback immediately following the intervention and up to 7 weeks after study entry (proximate feedback), as well as again 4 years later (long-term feedback). Thematic analysis was conducted to identify core themes, complemented by exploratory frequency and descriptive quantitative analyses comparing ApBM and sham conditions.

**Results:**

In total, 87 (ApBM: n=45; sham: n=42) of the 178 participants included in the randomized controlled trials provided proximate (7-week follow-up) feedback, and 63 (ApBM: n=33; sham: n=30) provided long-term (4-year follow-up) feedback; 104 participants contributed feedback at least once. Four overarching themes were identified, with the fourth emerging only in long-term reflections: (1) perceived treatment effects, spanning beneficial changes and a perceived lack of effects; (2) mechanisms of action, including presumed working mechanisms and impeding factors; (3) feedback on the training and study experience; and (4) attribution of effects to training-specific or external factors. Overall, participants engaged extensively in reflecting on potential mechanisms underlying the training, their relation to therapeutic outcomes, and opportunities for improving future interventions. Exploratory frequency analyses indicated that participants in the ApBM group more often suggested improvements to the training, whereas participants in the sham condition more frequently reported impeding factors, particularly difficulties understanding the training rationale. Quantitative training evaluations were generally more favorable for ApBM than for sham training, as reflected in a significantly higher overall evaluation score. In particular, ApBM received higher ratings for its perceived effectiveness in reducing smoking and its applicability to everyday situations.

**Conclusions:**

This study provides novel insights into how ApBM for smoking is experienced both shortly after training and years later. To our knowledge, this is the first study in the context of cognitive bias modification, including ApBM, to examine subjective experiences over a multiyear time frame. The findings highlight the importance of participants’ understanding of the training rationale and underscore the value of incorporating participants’ perspectives into the development and evaluation of ApBM interventions.

## Introduction

Cigarette smoking is highly addictive and remains prevalent despite its well-documented health risks. Globally, 1.18 billion people report regular smoking [1], with approximately half meeting diagnostic criteria for nicotine dependence [2]. Although the majority of individuals who smoke express an intention to quit [3], access to smoking cessation treatment remains limited to a small proportion of those in need. According to the World Health Organization [4], only about 30% have access to comprehensive smoking cessation services. Moreover, even among individuals who receive treatment, relapse rates remain substantially high [5,6]. Consequently, the development of feasible and easily accessible interventions that can complement and improve existing treatments represents a key priority not only in nicotine and tobacco research but also in public health.

One promising approach in this regard is the integration of therapeutic interventions into digital formats. In the context of smoking cessation, a growing number of mobile and computerized interventions have been developed, with evidence suggesting that incorporating cognitive behavioral therapy principles, such as self-reflection and cognitive restructuring, can support smoking cessation compared with no or minimal support (see Chu et al [7] for a recent meta-analysis). However, given the persistently high relapse rates even following treatment [5,7], it is likely that, beyond reflective processes targeted in traditional psychotherapeutic approaches, more automatic, implicit cognitive processes contribute to the maintenance of smoking behavior.

Over the past 2 decades, a range of computer-based training interventions has been developed to modify such implicit processes implicated in maladaptive substance use (for reviews, see Kakoschke et al [8] and Vrijsen et al [9]). Collectively, these interventions are referred to as cognitive bias modification (CBM). Whereas cognitive bias assessment tasks require equally frequent responses to substance-related and substance-unrelated stimuli (eg, equally often approaching and avoiding smoking and nonsmoking pictures), CBM training paradigms use systematic stimulus-response contingencies (eg, always avoiding smoking and always approaching nonsmoking pictures) to counteract the bias (for a recent review, see Vrijsen et al [9]). Hence, unlike traditional psychotherapeutic approaches, which primarily target reflective processes to facilitate behavioral change, CBM aims to directly alter more automatic, implicit processes through repeated training. To date, several CBM variants have been developed that differ in the specific cognitive processes they aim to modify. For example, attention bias modification (ABM) aims to train attentional allocation away from substance-related cues and toward more adaptive alternatives, using paradigms such as spatial cueing or gaze-contingent training. CBM for interpretations (CBM-I) targets the tendency to interpret ambiguous stimuli in a disorder-congruent way by consistently reinforcing benign or substance-unrelated interpretations. Approach bias modification (ApBM), on the other hand, repeatedly trains individuals to make avoidance movements in response to substance-related cues and approach movements toward neutral or goal-consistent cues [9]. Indeed, the strongest evidence for CBM’s clinical effectiveness comes from ApBM in alcohol use disorder (AUD), particularly when applied as an adjunct to psychological therapy in structured clinical settings (see Pan et al [10] for a recent meta-analysis and Wittekind et al [11] for a review). Several randomized controlled trials (RCTs) have demonstrated reductions in relapse rates of up to 10% at 1-year follow-up [12-16], which has led to the inclusion of ApBM in national treatment guidelines for AUD in Germany [17] as well as Australia [18].

Since then, this approach has been extended to other consumption-related behaviors, including nicotine use [19-23]. Although several studies have reported promising effects, such as increases in the number of abstinent days [19] or reductions in daily cigarette consumption following training [20,21], null findings have also been observed [23], and overall effect sizes remain modest (for a meta-analysis, see Boffo et al [24]). While multiple factors may contribute to this inconsistency, including differences in training design, participant characteristics, and outcome variables, participants’ subjective experiences of the training are likely to play an important yet underexplored role in ApBM effectiveness. This is particularly relevant given that multiple training sessions are required to induce reliable and lasting changes in cognitive biases [12,25], which may place a considerable burden on participants and affect adherence. Consequently, training acceptance and motivation are critical determinants of successful CBM implementation [26]. Yet, systematic qualitative investigation of participant experiences with CBM, particularly ApBM for smoking, remains limited. Evidence from the few available studies suggests that CBM tasks are often experienced as monotonous and repetitive [27] and that participants frequently report difficulties in understanding the relevance of the training for their behavioral goals [28]. Such perceptions are likely to undermine training motivation and engagement and may therefore partially account for the inconsistent clinical outcomes observed in the literature.

This notion is supported by evidence from related CBM domains. For example, in the context of ABM for social anxiety, one study showed that patients’ perceptions of the training predicted both bias reduction and subsequent symptom improvement [29]. Systematic research on subjective experiences with CBM in general, and ApBM in particular, remains scarce in the context of substance use, although a small number of studies suggest that participants’ perceptions may be important in this domain as well. For example, in an implementation study on ApBM for AUD, Schenkel et al [30] assessed training evaluation among alcohol dependent inpatients and found that although the paradigm was rated predominantly positive, most patients (60%) did not believe that the training was effective in reducing relapse rates, and only a minority (29%) perceived a transfer to everyday behavior. Extending these findings, a qualitative study investigating a combined ABM and ApBM intervention for problematic gaming behavior reported that negative training experiences, including perceptions of excessive duration and tediousness, might have accounted for extremely high attrition rates of up to 90% [31]. Thus, participants’ perceptions of CBM procedures are likely to contribute to both training uptake and effectiveness, underscoring the need for more systematic investigation of these perceptions and for strategies to improve them.

In line with this objective, recent advances in training delivery have aimed to enhance training acceptance by embedding ApBM in novel digital formats, including virtual reality (VR) and smartphone apps (for a review, see Forman et al [26]). These approaches are assumed to increase engagement and perceived relevance, as training can be conducted in immersive or consumption-related contexts. Supporting this notion, 2 proof-of-principle trials in the context of anxiety translated the principles of ABM [32] and CBM-I [33] into VR environments and reported high levels of enjoyment, motivation, flow, and presence. However, both studies were limited by small sample sizes, single-session training designs, and relatively short follow-up periods of up to 6 weeks. Besides, a mixed methods study combining internet-based ApBM and CBM-I for hazardous alcohol use and social anxiety reported overall satisfaction, but a need for a more compelling training rationale [34]. Similarly, a feasibility study on an app-based ApBM intervention for problem drinking found that in addition to positive feedback regarding usability and intuitiveness of app design, participants criticized the intervention as repetitive and insufficiently personalized [35]. In the context of ApBM for smoking, 2 larger RCTs were recently conducted by our research group, both including multiple training sessions and follow-up assessments. These studies demonstrated that ApBM delivered via VR [36] or a smartphone app [37] led to greater smoking reduction compared to respective control conditions. To date, however, little is known about how these novel delivery formats are perceived by individuals motivated to quit smoking, and whether common challenges associated with CBM, such as perceived monotony or a lack of meaningfulness, remain an issue of concern. Moreover, there are no long-term studies examining how such novel training approaches are perceived over extended periods of time or the extent to which they are retrospectively regarded as useful in the longer-term process of behavior change. This is particularly important, as research on psychotherapy outcomes indicates that patients’ perceptions of treatment may evolve over time, with important implications for sustained behavioral change and well-being. For example, Malkomsen et al [38] demonstrated that patients who were initially uncertain about the benefits of their psychotherapeutic treatment reported more positive views 3 years later. Such shifts may be particularly relevant given evidence that therapy perceptions influence whether learned skills are applied after treatment termination [39].

Against this background, this study examined subjective training experiences reported by participants from 2 RCTs that used VR-based [36] and app-based [37] ApBM for smoking cessation. The aims of the study were threefold: (1) to identify themes reflecting participants’ experiences with the training, (2) to examine how participants evaluate novel delivery formats of ApBM for smoking, and (3) to explore whether and how training perceptions change or remain stable over the long term. More specifically, regarding the first aim, we explored the extent to which emergent themes correspond to those identified in previous qualitative studies on CBM and psychotherapy more broadly, as well as whether study-specific themes arise in the present context. Regarding the second aim, we examined whether digitally delivered ApBM are associated with engagement and motivation and/or whether these novel formats introduce distinct challenges (eg, technical issues). Regarding the third aim, we investigated whether long-term reflections become more general over time or whether participants report additional or qualitatively different insights compared to their immediate evaluations. To answer these research questions, participants were invited to provide open-ended feedback immediately following the intervention and again approximately 4 years after study completion. Thematic analysis was used to identify core themes associated with the training principles. To contextualize and complement participants’ subjective accounts, descriptive information on smoking-related outcomes and quantitative training evaluations has been additionally assessed.

## Methods

### Ethical Considerations

This trial was approved by the local ethics committee of the University of Siegen (reference numbers ER_16_2018 and ER_18_2018) and was conducted in accordance with the Declaration of Helsinki and Good Clinical Practice guidelines. Participation was voluntary, and participants were able to withdraw their consent for participation at any time. Participants did not receive financial compensation for their involvement in the research.

To ensure privacy and confidentiality, all study data were stored on secure servers of the University of Siegen and were accessible only to authorized members of the study team. Participants were assigned a unique identification number, and all research data were stored and analyzed using these codes. A separate password-protected file linking participant names to identification numbers was maintained exclusively for study administration and follow-up purposes and was stored separately from the research data.

### Trial Procedure

This study examines subjective experiences with either a VR-based or app-based ApBM for individuals motivated to quit smoking. Quantitative outcomes related to training effects and clinical efficacy of these interventions have been reported previously (see Machulska et al [36,37]). For all participants, a brief smoking cessation intervention constituted treatment as usual (TAU), followed by a 14-day training program delivered either in VR or via a smartphone app. Participants randomized to the experimental group (EG) were trained to respond to smoking-related cues with avoidance movements and to alternative cues with approach movements. Participants in the control group (CG) received a sham training in which no systematic contingency between stimulus content and response movement was implemented.

In the app-based ApBM trial [37], an additional control condition was implemented, which received the TAU but was not given access to the training app during the main study period (baseline to short-term follow up). Consequently, feedback from this group is not presented in this analysis.

To explore participants’ subjective experiences, open-ended feedback was collected immediately after the training period (posttest), 7 weeks after baseline (short-term follow-up), and approximately 4 years after baseline (long-term follow-up). Notably, the posttest and 7-week follow-up assessments were both conducted within the active study phase of the original RCTs. Accordingly, both time points are considered to reflect participants’ proximate experiences and evaluations, rather than fully naturalistic, longer-term reflections. Based on this notion and to increase the amount of available qualitative data per time point, feedback from the posttest and short-term follow-up assessments was combined. Accordingly, these analyses distinguish between proximate feedback (posttest and 7-week follow-up) and long-term feedback (4-year follow-up).

Posttest and short-term follow-up assessments were conducted on-site at the laboratory. After completing the quantitative questionnaire battery, participants were invited to provide open-ended feedback in written form via the survey platform LimeSurvey. At the long-term follow-up, feedback was collected remotely. To maximize response rates, 2 complementary approaches were used. First, participants were contacted by telephone and, following a brief assessment of current smoking behavior, were invited to share open-ended feedback regarding the training and/or their overall study experience. In these cases, study assistants conducting the interviews documented participants’ responses verbatim. Second, all participants received an email invitation to complete an online survey, which also included an open-ended response field to allow for more general reflections on study participation.

### Participants

All participants took part in 1 of 2 RCTs investigating the effectiveness of a VR-based [36] or app-based [37] ApBM for smoking. Eligibility criteria for those RCTs included being at least 18 years of age, smoking a minimum of 6 cigarettes per day for at least 6 months prior to study entry, and reporting an intention to quit smoking within the subsequent 6 months. Exclusion criteria comprised a current substance use disorder other than nicotine, current psychiatric illness, insufficient proficiency in German, uncorrected visual or auditory impairments, or dyschromatopsia. In addition, participants who dropped out prior to the first training session or failed to become aware of the training contingencies in the EG were excluded from data analyses. This resulted in a total sample of 178 participants (n_EG_=87; n_CG_=91), of whom 96 received the VR-based training and 82 the app-based training. Overall, 104 of the 178 participants provided feedback at the proximate or long-term assessment. Of these, 54 had been randomized to ApBM training (n_VR_*=*35; n_App=_19) and 50 to sham training (n_VR_*=*29; n_App=_31). No differences were observed between participants who provided feedback and those who did not with regard to condition (*χ*^2^_1_=0.94; *P*=.34). However, participants in the VR trial were more likely to provide feedback than those in the app trial (*χ*^2^_1_=5.83; *P*=.02). The sample was evenly distributed by gender (52/104, 50% female), with age ranging from 22 to 67 years (mean 48.65, SD 11.73).

### Interventions

The interventions used in the 2 initial RCTs are briefly described below. Please refer to the original studies [36,37] for more detailed information.

#### TAU: Brief Smoking Cessation Intervention

Prior to the baseline assessment, all participants took part in a brief smoking cessation intervention (TAU), administered by a trained psychologist. The session was conducted in small groups of up to 4 individuals and lasted about 90-120 minutes. The following topics were covered by the intervention: psychoeducation, including reflective and impulsive determinants of smoking maintenance vs cessation, motivational support for smoking cessation, instructions to self-monitor daily smoking, and bibliotherapy through the provision of a self-help book.

#### Approach Bias Training Paradigm

##### Overview

Each training variant was based on the traditional joystick training, in which different pictures are presented consecutively on a computer screen, and participants are required to perform push (avoidance) or pull (approach) movements by means of a joystick attached to the computer. Upon a push movement, images shrink in size, creating a sense of avoidance, whereas upon a pull movement, images grow, creating a sense of approaching (see Rinck and Becker [40], for a description of the original task). This training principle was translated into 2 novel delivery modalities: a VR format in the first RCT [36], where participants executed push and pull movements with their own arms that were visible in the virtual environment, and a smartphone app in the second RCT [37], where participants performed upward (avoidance) and downward (approach) swiping movements on a touchscreen. All participants received the same cover story stating that the training aimed to target automatic processes involved in smoking by strengthening more controlled responses to smoking-related stimuli. In addition, it was communicated that this approach was expected to enhance self-control over smoking behavior in everyday contexts.

##### VR Training

The virtual environment was situated within an office setting, in which smoking-related and smoking-unrelated objects consecutively appeared on a virtual desk. Objects were either blue- or red-bordered. An indirect task instruction was used as participants were instructed to ignore object content and respond to border color only (ie, push red-bordered objects and pull blue-bordered objects). In the EG, all smoking-related objects were bordered in red, which required an avoidance movement, whereas all smoking-unrelated objects were bordered in blue, which required an approach movement. Hence, a 100%-contingency between object content and response movement was used. In the CG, participants performed a sham training version that closely resembled the VR-ApBM condition, except that there was no contingency between object content and response format. Each session took approximately 10-15 minutes to complete.

##### Smartphone App Training

Smoking-related and smoking-unrelated pictures appeared consequently at the center of a mobile screen. Pictures were either rotated 3° to the left or 3° to the right. An indirect task instruction was used as participants were instructed to ignore picture content and respond to image rotation only (ie, swipe-up pictures rotated to the right and swipe-down pictures rotated to the left). In the EG, all smoking-related pictures were rotated to the right and had to be swiped up (avoidance), whereas all smoking-unrelated pictures were rotated to the left and had to be swiped down (approach). As with the original ApBM training, a zooming feature was introduced to create a visual sense of avoiding vs approaching the image. In the CG, participants performed a sham training version that closely resembled the app-ApBM condition, except that no contingency between picture content and response format was introduced. Each session took approximately 5 minutes to complete.

### Assessments

#### Qualitative Data

Participants were invited to provide open-ended feedback in response to the question: “Do you have any feedback or comments about the training?” Responses were assessed at 3 time points: online immediately after the training interval (posttest) and 7 weeks after baseline (short-term follow-up), which were combined to represent proximate feedback, as well as online and via telephone approximately 4 years after baseline (long-term follow-up), which was combined to represent long-term feedback.

#### Quantitative Data

##### Overview

To aid interpretation of the qualitative findings, basic smoking-related and training-specific variables assessed at proximate and long-term time points are summarized below. For a comprehensive description of all measures administered in the original trials, please refer to the published study protocols [41,42] and original reports [36,37].

##### Smoking-Related Data

In addition to demographic information (ie, age and sex), participants provided smoking-related information, including (1) the duration of nicotine use (in years), (2) 7-day point abstinence, (3) number of daily smoked cigarettes, and (4) degree of nicotine dependence as measured with the Fagerström Test for Nicotine Dependence (FTND) [43] (German version [44]), with scores ranging between 0 (no/very weak dependence) and 10 (very strong dependence).

##### Training-Related Data

The number of completed training sessions was recorded either by study assistants (VR-based training) or automatically within the training app (app-based training). At posttest, participants additionally completed a brief training evaluation questionnaire consisting of 9 dichotomous items (0=no, 1=yes) assessing perceived ease (“The training was easy to perform.”), motivation (“It was difficult to motivate myself to complete the training.” [reversely coded]), clarity (“The training setup was clear.”), and subjective efficacy (“The training is beneficial in reducing smoking.”) of the training. Item responses were summed to yield a total evaluation score ranging from 0 to 9, with higher values indicating more positive evaluations of the training.

### Data Preparation and Planned Analyses

To explore participants’ perspectives and potential group differences in subjective training experience, we conducted a thematic analysis of the proximate and long-term open-ended responses. Three independent coders (AM, ES, and JB) followed an inductive, stepwise procedure for psychological thematic analysis according to Braun and Clarke [45,46]. All coders first reviewed the initial 20% of responses and generated codes from the data. They then met to compare codes, resolve discrepancies, and agree on a coding scheme. The remaining responses were coded using this scheme. Codes were subsequently grouped into higher-order subthemes and organized into overarching themes. Finally, the coding framework and theme assignments were reviewed by all coders and adjusted where necessary. Because the thematic analysis was exploratory, no a priori hypotheses were specified. Quotations presented in the Results were translated from German to English and checked for translation accuracy. To complement the qualitative analysis, we additionally quantified how often each theme and subtheme was mentioned and explored whether frequencies differed between the EG and CG using chi-square tests. Prior to quantification, the coders convened to ensure consistent application of the final coding framework across all participants. This procedure was undertaken to guarantee the reliability and validity of the theme assignment for the frequency-based analyses.

In addition, quantitative data regarding demographic variables, smoking-related behavior, and training-specific information are presented descriptively (means, SDs, and proportions) across conditions and time points to provide contextual information for the qualitative thematic analysis.

## Results

### Participants’ Characteristics

Participants’ characteristics with regard to demographic and smoking-related data are provided in Tables 1 and 2. As can be seen, the sample consisted of long-term smoking individuals who had been smoking for almost 30 years. The average consumption at baseline was approximately 19 (SD 6.59) cigarettes per day, with a moderate nicotine dependence (FTND score: mean 4.85, SD 2.12). Although descriptively, participants in the CG completed slightly more training sessions than those in the EG (CG: mean 9.1, SD 6.53 vs EG: mean 7.7, SD 5.27), short-term smoking-related outcomes improved to a similar extent in both groups. At the 4-year follow-up, descriptive differences between groups were observed for abstinence (30/48, 63% in the EG vs 13/38, 34% in the CG), daily cigarette consumption (EG: mean 6.19, SD 10.11 vs CG: mean 9.26, SD 9.36), and degree of nicotine dependence (EG: FTND: mean 1.29, SD 2.45 vs CG: mean 2.41, SD 2.80). Please note that these values are reported for contextual reasons only. Formal statistical analyses of smoking-related outcomes are presented in the primary RCT reports [36,37], with additional long-term analyses reported elsewhere [47].

**Table 1:** Sample sizes for overall participants and study groups across assessment time points.^a^

Variable	Overall (n=104), n	EG^b^ (ApBM^c^; n=54), n	CG^d^ (sham; n=50), n
Baseline	104	54	50
Posttest	100	51	49
Short-term (7-week) follow-up^e^	87	45	42
Long-term (4-year) follow-up	86	48	38

^a^Sample sizes were identical across outcome measures (abstinence and daily cigarette consumption).

^b^EG: experimental group.

^c^ApBM: approach bias modification.

^d^CG: control group.

^e^For nicotine dependence (Fagerström Test for Nicotine Dependence [43]) at the 7-week follow-up, data were available for 82 participants (EG=44 and CG=38) because scores could not be obtained from 5 participants.

**Table 2:** Demographic characteristics and smoking-related outcomes for the overall sample and study groups.

Variable		Overall	EG^a^ (ApBM^b^)	CG^c^ (sham)
**Demographic characteristics**				
	Age (years), mean (SD)	48.65 (11.73)	48.35 (12.98)	48.98 (10.33)
	Gender (female), n (%)	52 (50)	26 (48)	26 (52)
	Duration of nicotine use (years), mean (SD)	28.97 (12.08)	28.54 (12.85)	29.44 (11.29)
	Number of training sessions, mean (SD)	8.36 (5.92)	7.70 (5.27)	9.06 (6.53)
**Abstinence, n (%)**				
	Baseline	0 (0)	0 (0)	0 (0)
	Posttest	20 (20)	12 (24)	8 (16)
	Short-term (7-week) follow-up	21 (24)	11 (24)	10 (24)
	Long-term (4-year) follow-up	43 (50)	30 (63)	13 (34)
**Daily cigarette consumption, mean (SD)**				
	Baseline	18.93 (6.59)	19.69 (6.98)	18.11 (6.10)
	Posttest	10.25 (7.82)	9.88 (8.45)	10.63 (7.17)
	Short-term (7-week) follow-up	9.13 (6.86)	9.23 (6.98)	9.02 (6.81)
	Long-term (4-year) follow-up	7.54 (9.85)	6.19 (10.11)	9.26 (9.36)
**Nicotine dependence (FTND^d^ score), mean (SD)**				
	Baseline	4.85 (2.12)	4.91 (2.33)	4.78 (1.89)
	Posttest	3.04 (2.62)	3.00 (2.87)	3.08 (2.35)
	Short-term (7-week) follow-up	2.45 (2.36)	2.52 (2.49)	2.37 (2.24)
	Long-term (4-year) follow-up	1.78 (2.66)	1.29 (2.45)	2.41 (2.80)

^a^EG: experimental group.

^b^ApBM: approach bias modification.

^c^CG: control group.

^d^FTND: Fagerström Test for Nicotine Dependence [43]; scale: 0-10.

### Quantitative Training Evaluation

Table 3 summarizes participants’ quantitative responses to the training evaluation questionnaire at posttest. Overall, participants were satisfied with the training. The vast majority of participants reported that the training was clearly structured (97/99, 98%) and easy to perform (93/99, 94%), and most (83/99, 84%) indicated that they would recommend it for further use. Approximately three-quarters of participants perceived the training as helpful in reducing cigarette smoking, and 68% (67/99) reported no difficulties in motivating themselves to complete the sessions. Lower levels of agreement were observed for items referring to broader effects: only 37% (37/99) of participants endorsed the view that the training may contribute to abstinence, and 47% (47/99) believed it can be applied to everyday situations.

Although the overall pattern was similar across conditions, participants in the EG evaluated the training more positively than those in the CG, as indicated in a significantly higher training evaluation sum score (t_97_=3.08; *P*=.003). Specifically, participants in the EG indicated to have noticed more overall positive effects of the training (EG: 37/50, 74% vs CG: 24/49, 49%; *χ*^2^_1_=6.55; *P*=.01). In addition, a greater proportion of participants in the EG believed that the training was beneficial in reducing smoking (42/50, 84% vs 30/49, 61%; *χ*^2^_1_=6.47; *P*=.01) and could be transferred to everyday situations (30/50, 60% vs 17/49, 35%; *χ*^2^_1_=6.34; *P*=.01).

The training perceptions and between-group differences suggested by these quantitative findings are examined in greater depth in the qualitative analyses below.

**Table 3:** Training evaluations at posttest.^a^

Item	Overall	EG^b^ (ApBM^c^)	CG^d^ (sham)	*P* value
The training setup was clear, n (%)	97 (98)	50 (100)	47 (96)	*.*15
The training was easy to perform, n (%)	93 (94)	47 (94)	46 (94)	.98
I have noticed positive effects from the training, n (%)	61 (62)	37 (74)	24 (49)	*.*01
I have made progress in my reaction times during the training, n (%)	65 (66)	36 (72)	29 (59)	*.*18
The training is beneficial in quitting smoking, n (%)	37 (37)	22 (44)	15 (31)	*.*17
The training is beneficial in reducing smoking, n (%)	72 (73)	42 (84)	30 (61)	*.*01
Training can be applied to everyday situations, n (%)	47 (47)	30 (60)	17 (35)	*.*01
It was difficult to motivate myself to complete the training, n (%)	32 (32)	14 (28)	18 (37)	*.*35
The training should continue to be used, n (%)	83 (84)	44 (88)	39 (80)	*.*26
Sum Score (0-9)^e^, mean (SD)	6.28 (2.03)	6.88 (1.93)	5.67 (1.96)	.003

^a^Group differences regarding proportions were analyzed using chi-square tests; group differences in the sum score were analyzed using a 2-sample *t* tests. All *P* values are 2-tailed.

^b^EG: experimental group.

^c^ApBM: approach bias modification.

^d^CG: control group.

^e^This row indicates the sum score (0-9) for all items answered with “yes” (coded as 1), except for the item “It was difficult to motivate myself to complete the training,” which was reversely coded.

### Thematic Analysis of Participants’ Feedback on the Training

#### Overview

**Figure 1:**
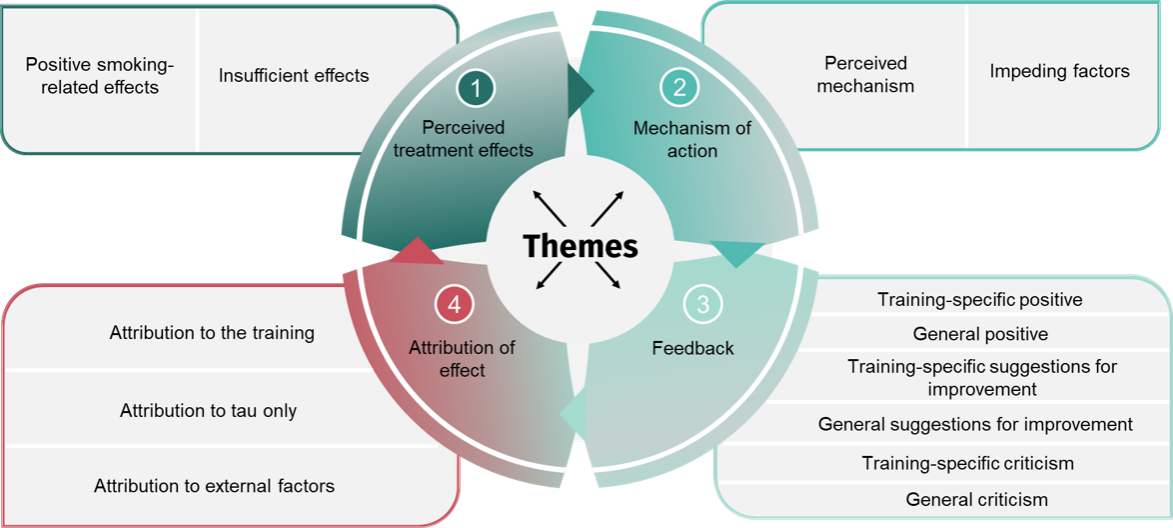
Overview of the thematic analysis.

Overall, the analysis yielded 4 overarching themes: *perceived treatment effects*, *mechanisms of action*, *feedback on the intervention*, and *attribution of effect*, each comprising several subthemes. Whereas the first 3 themes appeared at both time points, the fourth theme (attribution of effect) was only evident in the 4-year follow-up assessment. A comprehensive overview of all themes and subthemes is presented in Figure 1.

#### Participants’ Proximate Feedback

##### Overview

In total, 87 participants provided proximate feedback (posttest and/or 7-week follow-up). Of these, 45 were randomized to the EG, and 42 were in the CG. Example quotations are provided within the corresponding sections. In addition, each quote is accompanied by the participant ID, along with the intervention type (V=VR-based training; A=app-based training) and group assignment (1=EG; 0=CG). The analysis yielded the following overarching themes: *perceived treatment effects*, *mechanisms of action*, and *feedback on the intervention* (separate thematic analyses of the 4-year follow-up feedback yielded comparable results, with no meaningful differences in the thematic structure or content).

##### Theme 1: Perceived Treatment Effects

###### Overview

Participants described a broad range of perceived effects related to their participation, spanning positive smoking-related changes and reports of limited or absent effects.

###### Subtheme 1: Beneficial Smoking-Related Effects

Participants reported a variety of beneficial effects following the training. For instance, a notable number of participants described a reduced urge to smoke:

During the training, I did not feel any desire to smoke. I think that an intensive use of this training could indeed be beneficial and supportive.

134V1; post

This is great, I no longer have any desire to smoke.

101V1; FU

These subjective craving reductions were often accompanied by reports of a decreased daily cigarette consumption:

In general, I have noticed progress after training. I haven’t completely cut down on smoking yet, but I have significantly reduced my consumption.

89V1; post

Everything is going well; it has helped me to reduce consumption by half so far.

100V1; FU

Moreover, some participants reported having ceased smoking following training:

I managed to quit smoking, certainly with the help of the VR training.

7V1; post

Others provided nonspecific accounts of beneficial training outcomes. These participants noted general improvements attributed to the training, for example:

So far, it has been helpful and enjoyable.

100V1; post

###### Subtheme 2: Insufficient Effects

Alongside the positive effects outlined above, not all participants consistently reported beneficial or sustained outcomes from the study or the training. For instance, some noted only temporary improvements:

After the second session, I experienced a temporarily reduction in my urge to smoke. Unfortunately, this effect did not persist in the following sessions. My urge to smoke and smoking behavior have not changed.

50V1; FU

Similarly, some participants noted that reductions observed during the training phase subsequently dissipated:

During the two weeks of training, smoking behavior changed significantly, but after the training program ended, the effect wore off, mainly due to stressors in the personal and professional environment.

64A0; FU

Moreover, a subset of participants reported experiencing no discernible effects:

Unfortunately, it had no effect whatsoever on my smoking habits.

44V0; post

I couldn’t notice any change in myself.

42V0; post

Unfortunately, the training didn’t do anything for me at all.

129V0; post

To summarize, participants experienced a diverse array of smoking-related outcomes in relation to their study participation. While some participants reported a reduction in cravings, smoking, or complete abstinence, others exhibited only transient changes or no change at all.

##### Theme 2: Mechanisms of Action

###### Overview

This overarching theme comprises participants’ reflections on 2 distinct yet interconnected aspects of the training. First, it encompasses participants’ insights into how the training may have exerted its effects on smoking behavior. Second, it addresses participants’ perceptions of factors that may have limited or impeded the intended impact of the training.

###### Subtheme 1: Perceived Mechanisms of Action

Participants expressed a number of assumptions regarding the underlying mechanisms of action through which the training might have worked. Notably, some participants described processes that were closely aligned with the theoretical principles of ApBM. In particular, 1 participant attributed the training’s effects to the contingency between smoking-related stimuli and the push movement, stating:

In my opinion, it helps you think about other things than SMOKING. It has already helped me, and in stressful situations I was able to simply push smoking away.

53A1; post

Consistently, several participants reported that the training helped them to engage in more reflective smoking behavior or that the training disrupted habitual patterns:

Through training and the reason for training, I perceive the topic of smoking and when to smoke differently.

36V0; post

I thought that it worked well to break routines, especially when used specifically during the urge to smoke a cigarette.

25A1; post

While the aforementioned participants alluded to mechanisms of action in close alignment with the theoretical underpinnings of ApBM, other participants referred to more general mechanisms of action. For instance, some described that the training provided a helpful distraction from smoking:

It’s simply a good substitute for cigarettes at the beginning.

53A0; FU

The training is very helpful and shows me that I can become a non-smoker by ‘distracting’ myself from smoking objects or cravings.

48V0; FU

Moreover, for a subset of participants, simply dedicating time to the intervention was perceived as motivating. Notably, all of these participants took part in the VR-based training, where training sessions took place in a laboratory setting as opposed to the app-based training, which was conducted remotely.

Just going there had a positive effect on me.

15V0; FU

Overall, it was quite time-consuming to attend the 15-minute training sessions (travel time), but I also think that the considerable effort involved helped to motivate me to quit smoking. So, what seemed unpleasant and almost time-consuming at first was certainly what made it easier for me to quit in my case.

91V1; post

Lastly, some participants did not explicitly articulate assumptions about specific mechanisms of action, but expressed general confidence in the intervention:

The VR training certainly serves a purpose, even if I don’t recognize it.

53V0; post

Since I am not qualified to assess it neurologically, I trust that it [the training] helps with smoking cessation and remain curious.

3A0; post

###### Subtheme 2: Impeding Factors

Concurrently, participants identified several factors that impeded the training’s impact. These limiting factors refer to potential barriers to the implementation of ApBM within novel technological environments such as VR and app-based formats. Some factors perceived as limiting the training’s effectiveness were directly related to the training design itself. For instance, a number of participants indicated that the absence of a contingency between smoking-related stimuli and push movements limited the training’s impact on behavior. Notably, these participants were predominantly enrolled in the sham groups:

If I could just push all smoking-related objects away, the effect would perhaps be better?

1A0; post

I didn’t understand why I had to pull almost all the smoking-related objects toward me during the app training. In my opinion, it would have been better if I had pushed all the objects related to smoking away from me.

133A0; FU

Similar concerns were reflected in notes from participants in the EG, who perceived the approach bias assessment at posttest to measure training-specific bias change as a barrier that limited the intervention’s effectiveness:

After the last training session, the bias assessment from the pretest had to be repeated in order to evaluate reaction times. This involved pulling smoke objects towards me again. That ruined a lot of things. I found the effect very strong. In hindsight, I should have refused, following my first impulse.

79V1; post

In addition, many participants reported not fully understanding the purpose of the training, with some explicitly attributing this confusion to the lack of stimulus-response contingencies:

Since there was no need to consistently move smoke objects away from oneself or towards oneself, the purpose of the app was unclear.

3A0; post

Many images are pulled in one moment and pushed away the next. You’d think that makes little sense.

136A0; post

I didn’t understand the point of the exercises, because I don’t see how that’s supposed to help.

138A0; FU

Likewise, some participants noted that the indirect instruction to respond to a content-irrelevant feature diverted their attention from the stimulus content, which was associated with a lack of contingency awareness and, presumably, with a diminished training effect:

During the app training, I never saw the objects, only how the image was tilted.

107A0; post

I didn’t care if they [the stimuli] were smoke objects... I only paid attention to red and blue [border color].

86V0; post

In addition to impeding factors that were directly related to the training design, some participants referred to barriers in relation to aspects of the training implementation. For instance, technical difficulties were frequently reported, with some participants also describing these issues as diminishing their motivation:

Unfortunately, my mobile phone freezes and the images cannot be swiped away quickly enough. This reduces my motivation.

27A1; post

Such technical difficulties may provide an explanation for the insufficient or inconsistent training effects experienced by some participants. Finally, a number of participants identified everyday life factors as barriers to training adherence. For instance, external stressors were reported to have an impact on the ability of participants to complete training sessions:

I work in a three-shift system. If I haven’t done it [the training] in the morning before the afternoon shift, I tend to forget it.

84V1; post

Unfortunately, due to time constraints, I only completed the app training once. However, I noticed that my craving to smoke was suddenly reduced afterwards.

135A0; post

In my opinion, the planning/coordination was difficult for working people to implement due to the specified appointed times, etc.

42V0; FU

Closely related to this barrier, 1 participant observed that the training exhibited only limited transferability to everyday life:

In everyday life, “habits” still need to be broken.

48V0; post

In conclusion, participants indicated divergent assumptions regarding the mechanisms of action, spanning from more specific and theoretically grounded processes, such as actively pushing smoking-related cues away, to more general mechanisms, such as motivational effects and a general confidence in training effects. Notably, these specific assumptions were also reflected in perceived barriers, with participants in the sham condition reporting a desire to avoid smoking-related cues and some participants in the ApBM condition deploring reduced training effectiveness due to the posttest approach bias assessment, which removed the stimulus-response contingency. Moreover, technical problems and daily hassles were associated with limited training implementation and adherence to the training regimen. As accounts of factors impeding the training’s efficacy were given frequently, participants’ feedback on the intervention may provide insight into how to address such barriers in future applications of ApBM.

##### Theme 3: Feedback on the Intervention

###### Overview

This theme encompasses participants’ feedback on both the study and the training. Subthemes comprise positive feedback, suggestions for improvement, and criticism, which were all divided into both training-specific reflections as well as general study experiences.

###### Subtheme 1: Training-Specific Positive Feedback

Participants reported training-specific positive feedback, including overall satisfaction with the training, enjoyment, favorable impressions of the stimuli, ease of use, and a desire to continue engaging with the training. Moreover, certain participants emphasized the efficacy of the training in conjunction with the TAU prior to randomization:

Certainly not sufficient on its own for immediate smoking cessation, but definitely useful as a supplement to the [self-help] book, for example.

20A0; post

In addition, some participants underscored the training’s potential applicability to other domains:

The training is very helpful and shows me that I can become a non-smoker by ‘distracting’ myself from smoking objects or cravings. Yes, this training can also be applied to many other areas of life. Thank you very much for this.

48V0; FU

###### Subtheme 2: General Positive Feedback

Many participants additionally expressed satisfaction with the study, reporting gratitude and enjoyment. Furthermore, participants advocated for the continuation of the research:

Everything was perfect and very well organized. Thank you very much.

101V1; post

I really enjoyed it and it helped me reduce my cravings. Thank you very much!

26V0; post

###### Subtheme 3: Training-Specific Suggestions for Improvement

Beyond the positive feedback, participants provided ideas on several training-specific improvements. These included suggestions for technical enhancements, the use of direct instructions (ie, content-relevant feature training), and incorporating a larger variation in training scenarios and/or stimulus material:

Since the objects in the VR training are color-coded (red/blue), I find myself automatically looking only at the colors and not at the objects themselves. I think that if it were possible in terms of programming, I would stop assigning colors to the objects after the third training session so that you only have to focus on the object without the color dictating where the object has to go. I think that would be better for the effect of reacting and acting.

34V1; post

I would like to see more training sessions, possibly in different environments.

4V1; FU

Additionally, participants recommended decreasing the number of trials per session while increasing the number of training sessions:

The duration is a bit too long in some cases, but I would have liked to use it more often.

95A0; FU

Personally, I think the training with 200 images takes too long. [...]. I’d prefer the option of completing the training 2 times with 100 images instead.

112A1; post

I think 6 VR sessions are not enough.

76V1; FU

Consistently, participants also expressed a preference for an extended training interval:

The number of VR training sessions would have to take place at very regular intervals and over a longer period of time, then I would imagine it having an effect.

65V0; FU

Furthermore, participants consistently articulated the need for a better comprehension of the theoretical underpinnings of the training:

I would like to continue the app training and learn more about the background and scientific findings, ie, the specific goals and behavioral changes achieved through the training.

145A1; FU

Additionally, participants offered a plethora of recommendations regarding specific training application and/or implementation. For instance, some participants in the VR study assumed the training to be more effective when performed at home:

It would certainly be more effective if it took place more often, or if you could do it from the comfort of your own home.

66V1; post

VR training should be carried out daily, possibly programmed into an app.

26V0; FU

Other participants recommended integrating the training with additional interventions, such as:

I would also have liked to see more variety, eg, a stay in disgusting, dirty smoking rooms compared to a fresh flower meadow; [...]. Adding smells would certainly be successful.

13V1; post

Finally, participants put forth the notion of applying the training in specific situations and contexts. For instance, they noted its high use as a substitute for a cigarette:

It would be great if I could use the training more often to replace a cigarette; else, I tried to manage it without the app.

95A0; post

###### Subtheme 4: General Suggestions for Improvement

Participants additionally offered general suggestions to enhance the training’s impact on behavior. For instance, they expressed a desire for greater interaction and exchange with both fellow training participants and therapists:

Possibly [introduce] more training sessions and also discussions about [smoking] cessation

46V1; post

I would have liked personal conversations.

155A; FU

It would be beneficial to introduce group sessions to the VR training so that participants can exchange ideas with others. This helps to motivate.

49V1; FU

###### Subtheme 5: Training-Specific Criticism

Some participants also reported training-specific criticisms, highlighting its monotony and questioning its effectiveness as a stand-alone intervention:

I have the impression that the exercises should be modified with each additional training session, as in some cases they simply become monotonous.

27V1; post

After an initial learning phase, boredom sets in. A little more additional information would be desirable. Perhaps something encouraging, such as a counter for different things that improve over the course of quitting smoking, eg, how much CO2/tar/nicotine you have not inhaled/absorbed. An anonymous chat with like-minded people would also be nice

136A0; post

###### Subtheme 6: General Criticism

In addition, some participants expressed general criticism:

I cannot imagine that a long-term smoker would be motivated to quit smoking through VR training. At least not me, so I stopped after the first session!

33V1; post

Taken together, participants offered both positive feedback, including enjoyment and ease of use, and recommendations for improving the training. These recommendations included a higher training dosage, the implementation of direct task instruction, and the adaptation of the training for different contexts and situations. Some participants also voiced critiques of the study and training, particularly with respect to the effectiveness of the training as a stand-alone intervention.

Differences in perceived outcomes attributed to the training may reflect variations in how participants understood and experienced the training’s underlying mechanisms, as well as how these were shaped by training-specific features and individual or contextual factors. In particular, the stimulus-response contingency appears to play a central role in perceived meaningfulness and training efficacy, as several participants who experienced beneficial outcomes explicitly attributed these improvements to the stimulus-response contingency. Conversely, participants in the sham conditions linked insufficient effects to the absence of such a contingency. Furthermore, the mode of delivery also emerged as relevant: participants in the VR study described the training as motivating, enjoyable, and interesting, which aligns with the rationale for implementing ApBM in immersive environments. Likewise, participants in the app study appreciated the pictorial stimuli and expressed a wish to use the app more frequently in daily contexts. Building on this, participants’ descriptions of impeding factors and their suggestions for improvement provide further insights into why some individuals did not experience beneficial outcomes. Several participants reported technical problems during training, indicating that the implementation of technically innovative ApBM still needs to be refined. Others advocated for direct task instructions as well as a higher training variety and availability. The latter may be linked to reports of external stressors that diminished adherence to the training, thereby limiting its potential effectiveness. Finally, participants also recommended combining ApBM with other established interventions, assuming that they may benefit from multimodal approaches.

To complement the qualitative analysis, we quantified how often each theme was mentioned and explored whether frequencies differed between the EG and CG. These additional analyses are displayed in Table 4. Please note that due to the open response format, absence of mention does not indicate absence of experience. Inferential statistics should therefore be interpreted with caution and are used here only for exploratory purposes.

**Table 4:** Frequencies of reported themes and subthemes by group.^a^

Themes and subthemes		Immediate feedback: posttest and 7-week follow-up					Long-term feedback: 4-year follow-up			
		Overall, n	EG^b^ (ApBM^c^), n (%)	CG^d^ (sham), n (%)	*P* value	Overall, n		EG (ApBM), n (%)	CG (sham), n (%)	*P* value
Feedback provided		87	45 (29)	42 (24)	N/A^e^	63		33 (26)	30 (19)	N/A
**Theme 1: perceived treatment effects**										
	Beneficial smoking-related effects	25	17 (13)	8 (5)	.05	33		21 (19)	12 (9)	.06
	Insufficient effects	13	7 (5)	6 (5)	.87	15		6 (4)	9 (5)	.27
**Theme 2: mechanisms of action**										
	Perceived mechanisms of action	24	14 (7)	10 (6)	.45	9		6 (6)	3 (3)	—^f^
	Impeding factors	53	19 (10)	34 (16)	<.001	9		5 (4)	4 (4)	—
**Theme 3: feedback on the intervention**										
	Training-specific positive feedback	19	11 (4)	8 (5)	.54	12		8 (6)	4 (2)	.27
	General positive feedback	10	7 (5)	3 (2)	—	28		18 (12)	11 (7)	.16
	Training-specific suggestions for improvement	54	35 (22)	19 (10)	.002	2		0	2 (1)	—
	General suggestions for improvement	4	2 (2)	2 (0)	—	6		3 (3)	3 (2)	—
	Training-specific criticism	6	4 (4)	2 (0)	—	2		2 (2)	0	—
	General criticism	1	1 (2)	0	—	1		0	1 (1)	—
**Theme 4: attribution of the effect**										
	Attribution on the intervention	—	—	—	—	7		3 (2)	4 (3)	—
	Attribution on treatment as usual only	—	—	—	—	4		1 (1)	3 (2)	—
	Attribution on external factors	—	—	—	—	14		7 (7)	7 (5)	.84

^a^The number of mentions in relation to the virtual reality trial is shown in brackets. Differences between groups were analyzed using chi-square tests.

^b^EG: experimental group.

^c^ApBM: approach bias modification.

^d^CG: control group.

^e^N/A: not applicable.

^f^In cases where the expected cell frequencies fell below 5, no statistical tests were conducted.

As shown in Table 4, participants in the CG reported impeding factors significantly more often (*χ*^2^_1_=13.69; *P*<.001). A closer inspection of these comments indicated that most of these hindering factors centered on complaints about the lack of stimulus-response contingency and the perceived lack of meaningfulness of the training. Conversely, participants in the EG provided more suggestions for improving the training (*χ*^2^_1_=9.77; *P*=.002). Several interpretations may account for this pattern. For example, given that no group differences emerged in positive, training-specific feedback, the higher frequency of improvement suggestions in the ApBM groups may reflect a stronger cognitive engagement with the training procedures. Alternatively, it is possible that the more coherent and meaningful training experience in the EG facilitated participants’ ability to generate concrete suggestions for refinement, whereas the relative opacity of the sham condition may have limited such reflections. However, these interpretations remain speculative, as the study was not designed to systematically probe the underlying reasons for this difference. No other group differences reached statistical significance.

#### Participants’ Long-Term Feedback

##### Overview

In total, 63 participants provided open-ended comments at the final 4-year follow-up, of whom 33 were in the EG, and 30 were in the CG. The thematic structure largely mirrored the patterns observed for proximate feedback, encompassing *perceived treatment effects*, *mechanisms of action*, and *feedback on the intervention*. Each of these themes again comprised several subthemes comparable to those identified in the proximate feedback. Due to this substantial resemblance, detailed descriptions of the 3 recurring themes at the 4-year follow-up will not be presented here but are provided in the Multimedia Appendix 1. Notably, an additional overarching theme, *attribution of effects*, emerged uniquely at the 4-year follow-up, reflecting participants’ retrospective reflections on what they believed to have contributed to long-term changes. A comprehensive overview of all themes, subthemes, and their interrelations is presented in Figure 1.

##### Theme 4: Attribution of Effect

###### Overview

Responses at the 4-year follow-up revealed an additional theme related to participants’ attribution of treatment effects. Whereas earlier comments focused primarily on describing perceived changes, participants at the long-term follow-up engaged in a more elaborated meaning-making process by attributing their smoking-related outcomes—whether positive, limited, or absent—to specific causal sources. These attributions provide insight into how individuals retrospectively interpreted and contextualized their behavior change over an extended period.

###### Subtheme 1: Attribution to the Intervention

Several participants explicitly credited the training for their improvements, highlighting mechanisms or elements of the intervention they perceived as effective in supporting abstinence or reducing cigarette consumption:

Keep going. I am grateful for the app because it helped me to quit [smoking].

53A1; online assessment

Taking part in the study was very important for me, even though it took a long time until I finally managed to break away from nicotine. Taking part in the study also made me realize how harmful nicotine use is, which ultimately played a key role in my decision to quit smoking altogether. I would like to express my sincere thanks once again.

133V1; online assessment

While some participants referred broadly to the intervention as a whole, others explicitly attributed their outcomes to the combination of the training and TAU:

Everything was great; the book helped the most; the app was more of a supportive tool.

133A0; telephone assessment

###### Subtheme 2: Attribution to TAU Only

Some participants in the sham condition attributed their outcomes primarily to the support and guidance provided through TAU, namely, the bibliotherapy that was prescribed to all participants prior to randomization:

In retrospect, the book was very helpful; [I] only read it after the intervention had ended and then stopped.

57A0; telephone assessment

Because of the intervention, I succeeded in becoming a non-smoker. The book “Endlich Nichtraucher” [The Easy Way to Quit Smoking] contributed to this. The book was additionally handed out at the beginning of the study.

68V0; online assessment

###### Subtheme 3: Attribution to External Factors

A subset of participants attributed their smoking-related changes to factors outside the study context. These external attributions encompassed a range of factors that participants perceived as more decisive than the training itself. Medical issues were frequently cited as strong motivators for cessation:

[I had] a lung cancer surgery. Since then, I haven’t touched a cigarette.

115A0; telephone assessment

It was a very good experience, which unfortunately did not work for me, but I still managed it without any aids, because a serious illness that was related to smoking was the reason; and it’s just a matter of mindset and absolute willpower.

100V1; online assessment

In addition, some participants emphasized significant life circumstances such as the birth of a grandchild or the wishes of significant others as meaningful catalysts for change:

It didn’t work because of that [the study], but only later due to an illness and the birth of my grandchild.

63V0; telephone assessment

Some participants reported relying on other interventions, including additional cessation aids or self-initiated strategies outside the study protocol:

I did not smoke during the study, but unfortunately, I started again two weeks later. I made another attempt in mid-2023 with Fumexan smoking cessation therapy (injections into the ear) and it has worked quite well so far.

8V1; online assessment

Finally, 1 participant pointed to reasons unrelated to the training, suggesting that personal decisions or timing were more influential in determining their outcome than the intervention:

[I have] quit independently of the study 1.5 years ago.

93V0; telephone assessment

In summary, participants expressed a wide range of attributions regarding their long-term smoking-related outcomes. While some linked beneficial effects to the training and/or TAU, others pointed to additional or different factors outside the study context. Such attribution patterns underscore the complexity of smoking cessation processes and the varied meaning-making practices through which participants explained change long after the intervention had ended.

To quantify how often each theme was mentioned across groups, counts of reported themes and subthemes for the 4-year follow-up are also presented in Table 4. Similar to the proximate feedback, participants in the EG appeared to report more beneficial smoking-related outcomes; however, this difference did not reach statistical significance (*χ*^2^=3.52; *P*=.06). No group differences emerged for the remaining themes, including effect attribution (*P*_s_>.16).

## Discussion

### Principal Findings

#### Overview

This study sought to elucidate participants’ subjective experiences with 2 newly developed ApBM programs for smoking cessation, delivered either via VR or a smartphone app. In addition, the study examined factors perceived to contribute to beneficial or insufficient training effects, as well as retrospective reflections on the training that were obtained 4 years after study inclusion, alongside proximate feedback collected up to 7 weeks after the intervention. The thematic analysis identified four overarching themes, with the fourth emerging only in the long-term follow-up: (1) *perceived treatment effects*, ranging from positive smoking-related changes to a perceived lack of effects; (2) *mechanisms of action*, encompassing reflections on presumed working mechanisms as well as factors perceived as impeding effectiveness; (3) *feedback* on the training and the broader study experience; and (4) *attribution of effect*, referring to participants’ attempts to attribute behavioral change to training-specific or external influences.

#### Core Themes in Participants’ Experiences: Links to Prior Research and Novel Insights

With regard to our first research question, the overarching themes identified in this study are broadly comparable to those reported in previous qualitative studies investigating participants’ subjective perceptions of app-based psychotherapeutic interventions. For instance, Tudor-Sfetea et al [48] examined participants’ perceptions of a smoking cessation app based on cognitive behavioral therapy principles and identified themes such as “*application effects*,” comparable to the present theme of “*perceived treatment effects,*” as well as “*application content*” related to style, quality, and engagement, which partially overlaps with the current theme of “*mechanisms of action.*” At the same time, some themes appear to be specific to the present investigation, particularly “*attribution of effects.*” This difference may be attributable to the short-term assessment focus (1 week) in Tudor-Sfetea et al [48], compared to the additional long-term follow-up in this study.

More specifically, in the context of CBM, Kuckertz et al [29] investigated participants’ perceptions of 2 computer-based ABM interventions for anxiety and identified themes including “*perceived symptom change,*” “*mechanisms of action,*” “*acceptability and feedback,*” and “*barriers and facilitators to engagement.*” Similarly, Prior et al [34] asked adults with lived experience of hazardous alcohol use and social anxiety to evaluate a combined web-based ApBM and CBM-I intervention and revealed themes related to “anticipated clinical value,” “motivation and engagement,” and “perceived usefulness.” Thus, it appears that similar aspects are reported across different CBM training programs, which are not solely specific to ApBM or the training delivery method used. Consistent with other studies, our findings indicate that participants engage in complex reflection on the intervention that is difficult to capture through quantitative assessment methods alone. In the context of psychotherapy research, such treatment expectancies and their perceived potential to contribute meaningfully to improvement constitute a well-established common factor to treatment efficacy [49] and have been proven to predict treatment outcome [50]. In this trial, this dynamic was most clearly reflected in Theme 1 (*perceived treatment effects*) and Theme 2 (*mechanisms of action*). Participants reported a range of perceived outcomes following the training, including reductions in craving, decreases in cigarette consumption, and complete abstinence. Importantly, these perceived outcomes were closely connected with reflections on how the training was thought to work. Anticipated mechanisms of action included the existence of a stimulus-response contingency (ie, smoke-push trials), the disruption of automated action tendencies, and the strengthening of reflective processes related to smoking behavior. While these reflections are broadly consistent with both dual-process accounts of addiction [51] and the theoretical rationale underlying ApBM [11,13], they should be interpreted as subjective explanations rather than evidence of actual mechanisms, as findings in this regard remain mixed. For example, training-specific bias change or mediation effects have not been consistently observed (for a review and a meta-analysis, see Boffo et al [24]), suggesting that additional processes, potentially not accessible to subjective reflection, may also contribute to ApBM effects and remain to be further elucidated in CBM research.

By contrast, a subset of participants in the sham training groups reported difficulties in making sense of the task, especially due to the absence of a clear stimulus-response contingency. This lack of perceived meaningfulness was often accompanied by frustration and, in some cases, explicitly linked to insufficient training effects. Such accounts may also help explain the group differences observed in quantitative training evaluation ratings.

Germane to this, several participants expressed a desire for a clearer and more coherent theoretical integration of the training rationale (see Theme 3: *feedback*). In this study, the brief smoking cessation intervention (TAU) explained the impact of reflective and impulsive processes on smoking behavior, thereby introducing the rationale underlying the training as a means to target the latter. In addition, a cover story was used to convey the basic logic of the training task. Thus, participants were provided with basic explanations of the training’s purpose, which is consistent with recommendations emerging from prior qualitative research (eg, [34]). Nevertheless, these findings suggest that this level of explanation may not be sufficient and that a more sustained integration of the training rationale is warranted. In this instance, Kuckertz et al [29] compared a traditional, minimally explained ABM intervention with an “enriched” version that included more detailed explanations of the training’s theoretical background. Contrary to expectations, however, qualitative analyses revealed that participants in both conditions perceived the explanations as insufficient. Rather than receiving a single explanation at the outset, participants expressed a preference for repeated reminders of the training rationale and opportunities to discuss the training with clinicians during the program. This resembles established psychotherapeutic practices, in which treatment rationales are revisited and linked to patients’ everyday experiences and goals, and suggests a potential avenue for enhancing the perceived credibility of CBM interventions.

#### Perceptions of Digital Delivery Formats

With regard to the second research question, another important issue emerging from this study concerns participants’ perceptions of the novel delivery formats used for ApBM, namely VR and smartphone apps. While a few participants described the training as repetitive or suggested that it could be improved through greater stimulus variety, which is consistent with earlier reports on CBM task perceptions [27], others reported high levels of motivation and engagement. At the same time, several participants noted technical difficulties, which in some cases undermined motivation and disrupted training adherence. These findings highlight that the implementation of novel technologies may entail trade-offs, particularly when technological infrastructures are still under development. Importantly, prior CBM research suggests that more technologically advanced training approaches do not necessarily translate into better training perceptions. For instance, studies incorporating gamification elements [15], personalized stimuli [31], or adaptive designs [34] have reported mixed results with regard to user engagement and acceptability. Accordingly, efforts to enrich ApBM interventions should be undertaken cautiously, ensuring technical feasibility, and should not come at the expense of other critical factors such as participant support and the integration of the training into a broader theoretical or therapeutic framework (see the Core Themes in Participants’ Experiences: Links to Prior Research and Novel Insights section above).

#### Correspondence Between Short- and Long-Term Feedback

Regarding the third research question, comparisons between proximate and long-term feedback indicated that participants’ perceptions of the training were largely stable over time, as the 3 overarching themes identified at short-term follow-up were still evident 4 years later. Notably, a fourth theme emerged at the long-term follow-up concerning the *attribution* of beneficial outcomes, most commonly abstinence. Over time, participants appeared to develop a need to make sense of their behavior change, attributing it either to the training itself or to broader life circumstances. These attributions were almost evenly distributed, with approximately half of the participants explicitly emphasizing the training and/or the overall study experience as the primary reason for abstinence, while the other half referred to health- or family-related factors. Given that attribution was not explicitly probed, it is noteworthy that some participants spontaneously cited the training as causal even after such a prolonged period. As attributing change to internal or treatment-related rather than external factors is considered important for sustaining therapeutic gains [52,53], future research should systematically examine attribution processes in CBM and explore how training-related attributions can be fostered, for example, by providing and repeatedly reinforcing a theoretically and empirically grounded training rationale aligned with participants’ goals (see the Core Themes in Participants’ Experiences: Links to Prior Research and Novel Insights section above).

### Principal Contributions

This study contributes to the literature in several ways. First, it expands prior qualitative work on CBM by examining participants’ training experiences over an extended follow-up, spanning several years. Such a long time frame is rare in both CBM and psychotherapy research. Moreover, a relatively high response rate was achieved overall (104/178 participants provided feedback at least 1 assessment time point). In addition to the dual-contact approach used at follow-up (ie, telephone and online contact; see Methods), features of the original RCTs may have supported sustained engagement, including the predominantly in-person study procedures, which likely facilitated personal contact, rapport building, and peer interaction. The somewhat higher response rate in the VR-based ApBM condition further suggests that the immersive, on-site training format may have enhanced participant engagement. Moreover, the trials were explicitly framed as investigations of novel digital smoking cessation interventions, with an emphasis on both short- and long-term experiences communicated during the active study phase, which may have increased commitment to participate in the long-term follow-ups.

Second, although the overarching themes broadly align with prior qualitative work on CBM and related digital interventions, these findings provide additional insight into how different training conditions are experienced. Notably, participants in the sham condition frequently reported difficulties understanding the training rationale and, in some cases, explicitly criticized the absence of a stimulus-response contingency. This finding is particularly relevant for CBM research, as it suggests that blinding may be challenging when control conditions closely resemble the active training but lack its core mechanism. Such control conditions may not function as neutral comparators but could, in some cases, evoke frustration or reduced credibility, potentially influencing outcomes. At the same time, sham training was not consistently evaluated more negatively in this sample, and prior meta-analytic evidence indicates that sham interventions may still produce meaningful effects, particularly at higher training dosages [24]. Together, these findings highlight the ongoing challenge of identifying appropriate control conditions in CBM research and suggest that alternative comparator designs may warrant further investigation.

Third, these findings suggest that participants’ perceptions of ApBM remain relatively stable over time, while also giving rise to retrospective processes of sense-making. In particular, participants reflected on whether sustained behavior change could be attributed to the intervention or to external factors. Due to the relatively small number of spontaneous attribution-related statements, it was not possible to examine predictors of attribution or their relationship with long-term outcomes. This represents an important avenue for future research.

### Limitations and Future Directions

Alongside notable strengths, several limitations warrant consideration, which highlight relevant directions for future CBM research. First, the relatively high overall response rate may reflect both sustained motivation for smoking cessation and a willingness to contribute to research in this area. Hence, findings may not fully generalize to less intrinsically motivated or less engaged populations. While ApBM is often assumed to be more effective in motivated individuals or clinical samples, systematic evidence on the role of motivation in CBM outcomes is still limited. Future work should, therefore, examine whether motivation moderates ApBM effects or whether motivational processes can themselves be targeted by CBM principles, an issue that remains largely unresolved in the current literature.

At the same time, perspectives from a notable subset of the sample remained unavailable, especially at the 4-year follow-up. Exploratory analyses comparing participants who provided feedback at any time point with those who did not on key demographic, intervention-related, and smoking-related variables indicated largely comparable groups. However, selective attrition and survivorship bias cannot be fully excluded. For example, it remains possible that participants who provided long-term feedback were those for whom the intervention was more salient (ie, who were particularly satisfied or dissatisfied), which may have influenced both the qualitative themes and the interpretation of descriptive outcome data. Relating to this, although participants were encouraged to provide both positive and negative feedback, and the thematic analysis identified a range of critical next to favorable perspectives, responses may still have been influenced by demand effects. Hence, incorporating multimodal assessments beyond self-report, such as behavioral indicators or momentary assessments, may be a fruitful direction for future research to reduce possible bias and enhance the validity of findings.

Third, the thematic analysis was based on unprompted, open-ended responses. As participants decided independently which aspects of their experience to report, the absence of a particular theme cannot be equated with the absence of the corresponding experience. This limitation may be particularly relevant for the theme of *attribution of effects*, which emerged only at the 4-year follow-up. Moreover, the open-ended question used to elicit feedback was phrased in broad terms. While some participants provided in-depth reflections on the training and its potential mechanisms, others offered more general evaluations, resulting in considerable variability in the specificity of responses. Future research could therefore benefit from targeted qualitative or mixed methods approaches—for instance, semistructured interviews—which would allow for a more systematic examination of attribution processes over time.

### Implications for ApBM Task Design and Implementation

These findings offer important implications for the design and implementation of future CBM interventions. These relate primarily to (1) the communication of the training rationale and management of expectancy effects, (2) the optimal dosage and contextual embedding of the training, and (3) specific design features of the training tasks.

First, participants’ requests for a stronger theoretical integration of the training, combined with greater therapeutic support, highlight the importance of how the training rationale is communicated. While many CBM trials deliberately minimize expectancy effects to enhance internal validity (ie, by providing limited information about the training’s rationale or theoretical background), implementation-oriented studies aiming to maximize clinical benefit may need to adopt a different strategy [54]. In clinical and medical settings, it is well established that providing patients with clear information about an intervention, along with conveying confidence and therapeutic rationale, can meaningfully influence outcomes [55]. Accordingly, greater emphasis on external validity in CBM research, including ApBM, may be warranted, particularly when interventions are delivered to clinical or affected samples. Such an approach may not only enhance training efficacy but may also be in better accord with participants’ expectations and preferences. Future work may, therefore, incorporate more transparent and evidence-informed rationales, for example, by referencing prior findings on ApBM effectiveness, to enhance engagement, perceived training relevance, and training-related effect attributions.

Second, participants frequently suggested increasing training dosage and embedding the intervention more strongly within supportive contexts, such as peer exchange. These suggestions are in line with both dual process accounts of addiction [51], which stress the importance of targeting both reflective and impulsive processes to contribute to sustained behavioral change, as well as empirical findings that show that multisession CBM as an add-on to regular treatment tends to be more effective than single-session or stand-alone trainings [24,25]. At the same time, the number and spacing of training sessions remain to be determined. In the context of ApBM for AUD, one study suggests that 6 training sessions may be optimal in terms of learning effects [25], which informed the design of the present interventions, particularly the VR-based training. However, further research is needed to establish optimal training dosage for ApBM in the context of smoking cessation. Furthermore, participant suggestions regarding context-sensitive training use (eg, during craving or before or after quitting attempts) align with ongoing developments in the field [22,56] and further highlight the potential value of involving participants in the iterative development of CBM interventions (see Prior et al [34], for an encouraging example of a participatory codevelopment approach for an ApBM + CBM-I online intervention). Taken together, task design decisions should integrate evidence-based mechanisms of ApBM with participants’ experiential insights regarding how the intervention is perceived to work, as optimizing outcomes may depend on aligning these complementary perspectives.

Third, participants’ feedback also pointed to specific design features of CBM tasks. In particular, some participants reported that the indirect task instruction (ie, react to a content-irrelevant feature) prevented them from paying attention to the stimulus content. As a result, they suggested that direct task instructions (ie, approach-avoid movements explicitly based on stimulus content) might enhance training effectiveness. Traditionally, CBM paradigms rely on indirect instructions, requiring participants to ignore stimulus content and respond instead to content-irrelevant features. Empirical evidence directly comparing instruction types is limited. Yet, one study contrasted content-relevant (emotional) vs content-irrelevant (color-based) instructions and showed that emotional stimulus features were still processed when being task-irrelevant [57], underscoring the main assumptions of CBM [50,58] and suggesting that indirect instructions are not inherently inferior despite participants’ reservations. Importantly, such findings also highlight that participant accounts can inform the refinement of intervention design, but that proposed adaptations require systematic empirical testing. At the same time, evidence suggests that training effects under indirect instructions may be enhanced by increasing participants’ awareness of the stimulus content [57]. Such findings show that participant-informed design adaptations, such as increasing the salience of contingencies without abandoning indirect paradigms, may represent promising avenues for future task adjustments but require systematic testing.

### Conclusions

To conclude, this trial demonstrates that participants’ subjective experiences constitute a central dimension of how ApBM for smoking is perceived and engaged with. By assessing both proximate (up to 7-week follow-up) and long-term (4-year follow-up) perspectives, the findings highlight that behavior change is embedded in an ongoing process of meaning-making that extends beyond the duration of the training. Future research should continue to place emphasis on participants’ perceptions and incorporate them into the development of ApBM protocols by adopting participatory and patient-centered approaches. Such efforts may help to enhance both the effectiveness and acceptability of the intervention by better aligning training procedures with participants’ needs and experiences.

## Appendix

Multimedia Appendix 1 Detailed information on the first three overarching themes—(1) perceived treatment effects, (2) mechanisms of action, and (3) feedback on the intervention—based on 4 participants’ long-term (4-year) follow-up responses.

## Abbreviations

ABM: attention bias modification
ApBM: approach bias modification
AUD: alcohol use disorder
CBM: cognitive bias modification
CBM-I: cognitive bias modification for interpretation
CG: control group
EG: experimental group
FTND: Fagerström Test for Nicotine Dependence
RCT: randomized controlled trial
TAU: treatment as usual
VR: virtual reality

## Data Availability

The dataset used and analyzed during this study will be made available at Open Science Framework [59].
