# Supplementary material for: Proximal and Long-Term Participants’ Perspectives on Approach Bias Modification for Smoking Cessation: Qualitative Study
Source: JMIR Hum Factors. 2026 Jun 22;13:e93238. doi: 10.2196/93238 (PMC13338674; doi:10.2196/93238)
Supplement: Multimedia Appendix 1 [file humanfactors_v13i1e93238_app1.pdf]

Supplemental Material Appendix

This section provides detailed information on the first three overarching themes (a. *perceived treatment effects*; b. *mechanisms of action*; c.) *feedback on the intervention*) based on participants' long-term (four-year) follow-up responses.

Participants' long-term feedback at the four-year follow-up

Theme 1: Perceived treatment effects

Similar to responses at the proximate assessment, participants described a broad spectrum of perceived treatment effects, ranging from sustained positive smoking-related changes to absent or insufficient effects. While several participants reported continued improvements or maintenance of earlier gains, others indicated that initial benefits diminished over time or that no beneficial effects occurred at all.

Sub-theme 1: Beneficial smoking-related effects

Several participants described meaningful reductions in craving as a lasting benefit of the intervention:

> *Participating in the therapy [study] certainly helped me [to quit], because I have now been smoke-free for over three years and have absolutely no desire to smoke anymore. I exercise a lot; that is now my way of balancing things out. [49V1; online assessment]*

In line with proximate feedback, some participants reported lower daily cigarette consumption:

> *Shortly after the end of the study, [I have] significantly reduced [smoking] (max. 3 cigarettes/day) [34V1; telephone assessment]*

Others reported complete abstinence:

Thank you for letting me participate in the smoking cessation program. I have been completely smoke-free ever since, and I am very happy about that! [15V0; online assessment]

Participants also provided non-specific positive evaluations of the intervention:

Very content with the result, good program [134V1; telephone assessment]

Sub-theme 2: Insufficient effects

Some participants reported experiencing only short-term effects:

During the study I did not smoke, but unfortunately, I started again two weeks afterwards. [8V1; online assessment]

Others reported no discernible treatment effects:

I really enjoyed taking part in the study but it did not change the effect on my smoking behavior. [10V1; online assessment]

Didn't help. [145A1; online assessment]

Cost-benefit considerations were also reflected during the long-term assessment, highlighting a perceived mismatch between effort and outcome for some participants:

A lot of effort for no result [53V0; telephone assessment]

Overall, long-term responses four years later closely echoed the proximate feedback findings: some participants maintained clear benefits, while others experienced only brief or no changes on smoking behavior, with some individuals in the VR study highlighting that the effort invested may not have justified the outcome.

Theme 2: Mechanisms of action

In line with findings from the proximate feedback assessments, this theme comprises two central aspects of the training: perceived mechanisms of action and factors hindering potential effects.

Sub-theme 1: Perceived mechanisms of action

Similar to the proximate feedback, one frequently mentioned mechanism involved the interruption of automatisms or habitual smoking patterns:

> *Helped to break habits; for example, not carrying cigarettes with oneself when leaving the house. [40V1; telephone assessment]*

Again, developing a more reflective smoking behavior was named as a mechanism of action:

> *Significantly increased [my] awareness of consumption behavior. [19V0; telephone assessment]*

> *Made me aware / created awareness [70V0; telephone assessment]*

For some, this increased awareness translated into better control over their consumption:

> *The training was interesting; it changed something in my awareness; I was able to control my consumption better. [4V1; telephone assessment]*

Sub-theme 2: Impeding factors

At the same time, participants also identified a range of impeding factors that limited the intervention's effectiveness. Some noted an absence of contingency during the posttest bias measurement, which reduced the perceived impact of the previous training tasks:

In the last virtual session, the concept had been changed; there [I] had to pull (cigarettes). [I] think this is why [I] started smoking again; prior to this, [I] have had to push away cigarettes and similar objects. [79V1; telephone assessment]

Others described a lack of perceived meaningfulness, stating that the exercises did not feel purposeful or sufficiently connected to their smoking behavior.

The purpose of the virtual training was not clear to me. Therefore, my motivation to participate was not sustained. [85V0; online assessment]

External pressures, such as stress or occupational demands, were also cited as interfering with the ability to benefit from the intervention:

One's own life has to be managed, what has been built up needs to be maintained, and on top of that, we are fully employed. Our own parents, however, are becoming 'old and difficult.' Elderly people are in a league of their own, and here we are challenged with a great deal of patience and responsibility. All of this combined with the fact that we are no longer as resilient as we were at 40. I am 58 and often reach my limits. [19V0; online assessment]

Additionally, several individuals reported a lack of abstinence motivation, indicating that they were not ready or willing to quit at the time of the intervention:

[...] I managed to stop smoking on my own much later. At the time [of study participation], I wasn't really mentally ready, yet. But I enjoyed it very much. [16V1; online assessment]

Finally, a few participants questioned the indication of VR-based training, suggesting that it may not be appropriate for certain issues, such as left–right discrimination difficulties, or that the VR-format felt more suitable for children or younger users:

Found it very alienating with the VR headset; discontinued after a few sessions → something like that is more for children, not for people who have been smoking for 40 years. [33V1; telephone assessment]

Theme 3: Feedback on the intervention

Participants' reflections during the fout-year follow-up period included extensive feedback on the intervention, largely echoing the themes identified in the proximate assessments. Their comments captured both appreciative perspectives and areas for refinement, addressing training-specific features as well as broader aspects of the study experience.

Sub-theme 1: Training-specific positive feedback

Participants offered a range of positive comments directed specifically at the training. Several described the program as interesting:

Not bad; the VR was very interesting, was good; [49V1, telephone assessment]

Others expressed satisfaction with the training, noting that the structure or content met their expectations or provided a meaningful experience. Additionally, some participants reported a willingness or desire to continue using the app or training beyond the study period.

Sub-theme 2: General positive feedback

Participants also offered a range of positive reflections that extended beyond the training itself. Several expressed that they were glad to have participated or that they enjoyed the experience, emphasizing the personal value they derived from taking part:

I really enjoyed taking part in the study. [10V1; online assessment]

It made a difference, [I am] satisfied and had also fun.[101V1, telephone assessment]

Some used the opportunity to encourage continuation of the study or training, signaling their belief in the program's potential benefits for others. Others conveyed overall satisfaction with the study, highlighting the organization, structure, or support provided throughout the process:

[I was] very satisfied with the support and contact. [58A0; telephone assessment]

Additionally, expressions of gratitude were common, with participants thanking the research team for the opportunity to participate or for the support received.

Once again, many thanks for allowing me to participate in the study. It helped me – though not immediately – to say goodbye to nicotine. All the best to you and your team. [48V0; online assessment]

Sub-theme 3: Training-specific suggestions for improvement

In addition, participants offered concrete suggestions for improving the training itself. A recurring recommendation, already noted at the proximate feedback, was the desire for a longer training interval, with participants assuming that a more extended period of engagement would provide more sustained support throughout the cessation process.

[I] found the program too short, not enough follow-ups, too abrupt. [29V0, telephone assessment]

Sub-theme 4: General suggestions for improvement

Participants also provided broader suggestions for enhancing the overall study experience. Several emphasized the value of more opportunities for exchange with other participants, noting that peer interaction could offer additional motivation, shared understanding, and social support:

Meeting in a group more often would be good (exchange of experiences). [12V0; telephone assessment]

Others expressed a desire for more intensive and individualized guidance suggesting that tailored support or closer contact with the study team might better address personal needs and strengthen engagement throughout the cessation process:

[I] would have liked more individual support, more individualized programs. [86V0; telephone assessment]

Sub-theme 5: Training-specific criticism

Training-specific criticism referred to perceiving the training as too simple:

Seemed a little simple [13V1; telephone assessment]

Not yet fully developed; easy to see through. [27V1; telephone assessment]

Sub-theme 6: General criticism

Finally, one code included general unspecified criticism:

It wasn't a good 'seminar,' didn't help much [86V0; telephone assessment]

To summarize the findings at the long-term follow-up assessment, participants' reflections covered familiar topics from earlier time points, including perceived treatment effects, mechanisms of action, and feedback on the intervention. As before, participants described a broad range of experiences, from sustained abstinence and increased awareness to limited or absent effects, as well as factors that supported or hindered behavior change.
